# Susceptibility of murine induced pluripotent stem cell-derived cardiomyocytes to hypoxia and nutrient deprivation

**DOI:** 10.1186/s13287-015-0057-6

**Published:** 2015-04-23

**Authors:** Andreja Brodarac, Tomo Šarić, Barbara Oberwallner, Shokoufeh Mahmoodzadeh, Klaus Neef, Julie Albrecht, Karsten Burkert, Matteo Oliverio, Filomain Nguemo, Yeong-Hoon Choi, Wolfram F Neiss, Ingo Morano, Jürgen Hescheler, Christof Stamm

**Affiliations:** Berlin-Brandenburg Center for Regenerative Therapies, Föhrer Str.15, Berlin, 13353 Germany; Center for Physiology and Pathophysiology, Institute for Neurophysiology, Medical Faculty, University of Cologne, Cologne, Germany; Department of Cardiothoracic Surgery, Heart Center, University Hospital Cologne, Cologne, Germany; Max-Delbrueck-Center for Molecular Medicine, Berlin, Germany; Max-Planck-Institute for Metabolism Research, Cologne, Germany; Department of Anatomy I, Medical Faculty, University of Cologne, Cologne, Germany; Deutsches Herzzentrum Berlin, Berlin, Germany

## Abstract

**Introduction:**

Induced pluripotent stem cell-derived cardiomyocytes (iPS-CMs) may be suitable for myocardial repair. While their functional and structural properties have been extensively investigated, their response to ischemia-like conditions has not yet been clearly defined.

**Methods:**

iPS-CMs were differentiated and enriched from murine induced pluripotent stem cells expressing enhanced green fluorescent protein (eGFP) and puromycin resistance genes under the control of an α-myosin heavy chain (α-MHC) promoter. iPS-CMs maturity and function were characterized by microscopy, real-time PCR, calcium transient recordings, electrophysiology, and mitochondrial function assays, and compared to those from neonatal murine cardiomyocytes. iPS-CMs as well as neonatal murine cardiomyocytes were exposed for 3 hours to hypoxia (1% O_2_) and glucose/serum deprivation, and viability, apoptosis markers, reactive oxygen species, mitochondrial membrane potential and intracellular stress signaling cascades were investigated. Then, the iPS-CMs response to mesenchymal stromal cell-conditioned medium was determined.

**Results:**

iPS-CMs displayed key morphological and functional properties that were comparable to those of neonatal cardiomyocytes, but several parameters indicated an earlier iPS-CMs maturation stage. During hypoxia and glucose/serum deprivation, iPS-CMs exhibited a significantly higher proportion of poly-caspase-active, 7-aminoactinomycin D-positive and TUNEL-positive cells than neonatal cardiomyocytes. The average mitochondrial membrane potential was reduced in “ischemic” iPS-CMs but remained unchanged in neonatal cardiomyocytes; reactive oxygen species production was only increased in “ischemic” iPS-CMs, and oxidoreductase activity in iPS-CMs dropped more rapidly than in neonatal cardiomyocytes. In iPS-CMs, hypoxia and glucose/serum deprivation led to upregulation of Hsp70 transcripts and decreased STAT3 phosphorylation and total PKCε protein expression. Treatment with mesenchymal stromal cell-conditioned medium preserved oxidoreductase activity and restored pSTAT3 and PKCε levels.

**Conclusion:**

iPS-CMs appear to be particularly sensitive to hypoxia and nutrient deprivation. Counteracting the ischemic susceptibility of iPS-CMs with mesenchymal stromal cell-conditioned medium may help enhance their survival and efficacy in cell-based approaches for myocardial repair.

## Introduction

Transplantation of cardiomyocytes (CMs) into injured myocardium has been shown to improve contractile function in animal models of heart disease [1,2]. With advances in genetic reprogramming technology, creation of induced pluripotent stem cells (iPSCs) and improvements in differentiation protocols, it is now possible to produce large quantities of patient-specific, autologous CMs (induced pluripotent stem cell-derived cardiomyocytes; iPS-CMs) *in vitro* [3,4]. However, low retention, survival, and engraftment of transplanted CMs in the ischemic heart greatly hamper clinical application of these cells [5]. Cell loss is dramatic even when cell types with high tolerance to ischemia, such as mesenchymal stromal cells (MSCs), are transplanted into infarcted myocardium [6]. A detailed understanding of the cellular response to ischemia-like stress is therefore necessary for improving the efficacy of cell-based myocardial regeneration. Structurally and functionally, iPS-CMs were shown to display properties of fetal or neonatal CMs [7-10]. In contrast to adult mature CMs, which depend on oxidative metabolism for ATP synthesis, immature CMs can generate ATP through glycolysis and should to be more resistant to hypoxia [11,12]. We therefore analyzed the response of murine iPS-CMs and their neonatal murine counterparts (N-CMs) to hypoxia and glucose/serum deprivation (GSD) *in vitro*, and also tested the potential of paracrine factors secreted from bone marrow-derived multipotent MSCs to protect iPS-CMs from deleterious effects of “simulated *in vitro* ischemia” [13,14].

## Methods

### Induced pluripotent stem cell differentiation and purification

**Figure 1:**
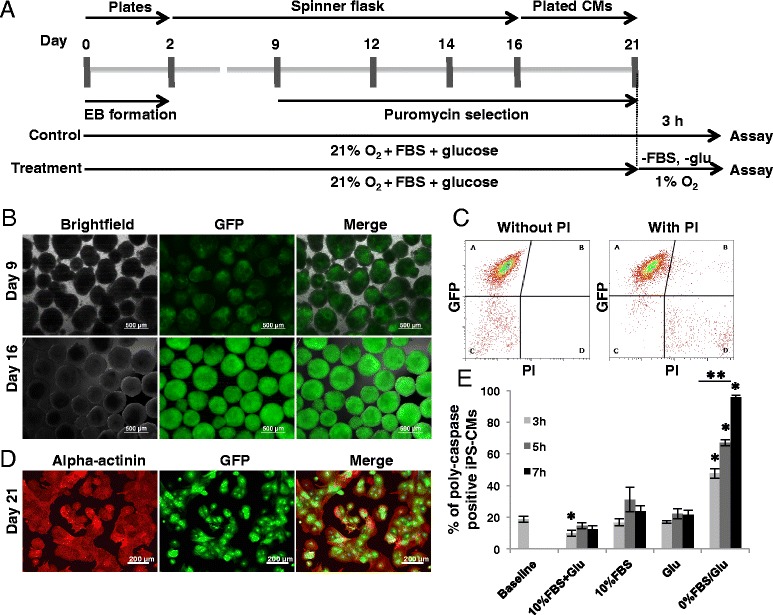
Differentiation of murine induced pluripotent stem cells (iPSCs) into cardiomyocytes (CMs). **(A)** Schematic representation of the experimental protocol. **(B)** Representative images of embryoid bodies (EBs) composed of a mixed cell population containing areas of GFP-expressing CMs on differentiation day 9. Purified cardiac clusters were generated by selection with puromycin until day 16. **(C)** Flow cytometry analysis of GFP-positive iPS-CMs after trypsinization of puromycin-selected cardiac clusters on day 16. **(D)** α-Actinin (red) staining of puromycin-selected CMs (green) on differentiation day 21. **(E)** Effect of hypoxia and glucose/serum deprivation on poly-caspase activity in iPS-CMs. The baseline level of poly-caspase active in iPS-CMs was determined in normoxia and full medium. *P ≤ 0.05, versus normoxia for individual datasets (Bonferroni test); **P < 0.001, overall by analysis of variance. FBS, fetal bovine serum; GFP, green fluorescent protein; Glu, glucose; PI, propidium iodide.

Murine iPSCs generated from 129S4/Sv4JaeJ x C57Bl/6 tail tip fibroblasts were generously provided by the Jaenisch group [15]. These iPSCs were genetically modified to express enhanced green fluorescent protein (GFP) and puromycin resistance genes under the control of an α-myosin heavy chain promoter as previously described for murine embryonic stem cells [16]. Undifferentiated iPSCs were grown on irradiated mouse embryonic fibroblasts (CellSystems, Troisdorf, Germany) in Dulbecco’s modified Eagle's Medium (DMEM) supplemented with 15% fetal bovine serum (FBS), 1% non-essential amino acids, 50 μM β-mercaptoethanol (all from Life Technologies, Darmstadt, Germany) and 1000 U/ml leukaemia inhibitory factor (LIF) (Merck Millipore, Darmstadt, Germany). Cardiomyocyte differentiation of iPSCs was performed as summarized in Figure 1A. One million iPSCs were incubated in a Petri dish on a horizontal shaker (60 rpm) in 14 ml differentiation medium composed of Iscove’s modified Dulbecco’s medium (IMDM), 20% FBS, 1% non-essential amino acids, 0.1 mM β-mercaptoethanol (all from Life Technologies) and 30 μg/ml ascorbic acid (Wako Chemicals USA Inc., Richmond, VA, USA). Two days after initiation of differentiation, embryoid bodies (EBs) were transferred into 250 ml spinner flasks (Integra Biosciences, Fernwald, Germany) at a density of 30,000 EBs per 200 ml differentiation medium. Since iPS-CMs expressed both enhanced GFP and puromycin resistance, the addition of 8 μg/ml puromycin (PAA, Cölbe, Germany) from differentiation day 9 until day 16 resulted in a highly pure population of GFP-positive and spontaneously contracting CMs. Fresh puromycin was added every second day. On differentiation day 16, iPS-CMs were dissociated with 0.25% trypsin-ethylenediaminetetraacetic acid (EDTA) and their purity was assessed by flow cytometry (FACS Calibur, BD Biosciences, San Diego, CA, USA). For all subsequent analyses, dissociated CMs were plated on fibronectin-coated plates and maintained for an additional 5 days as described below.

### Neonatal mouse cardiomyocytes

N-CMs were purchased from ScienceCell (Provitro, Berlin, Germany), where they were characterized by immunostaining with antibodies specific to smooth muscle actin, sarcomeric alpha-actinin, and tropomyosin. Their purity was estimated to be between 95 and 97%, with viability approximately 98%. N-CMs were cultured in medium that selectively promotes CM growth to preserve their purity and prevent outgrowth by non-CMs. It consisted of basal medium (cat. no.6201), 5% FBS (cat. no. 0025), 1% of specific CM growth supplement (cat. no. 6252) and 1% of penicillin/streptomycin solution (cat. no. 0503) (all from Provitro). At confluency, after 10 to 12 days in culture, cells were trypsinized and 40,000 cells/well plated on fibronectin-coated plates for subsequent analyses.

### Immunocytochemistry

On differentiation day 21, cells were fixed with 4% buffered paraformaldehyde (PFA) and permeabilized with 0.1% Triton X-100. Followed by blocking in 5% goat serum, cells were stained with anti-sarcomeric-α-actinin (clone EA-53, 1:400; Sigma, Sigma-Aldrich, Steinheim, Germany) in 0.8% bovine serum albumin/phosphate-buffered saline (BSA/PBS). After washing in PBS, incubation with anti-mouse-igG1 Alexa Fluor 555 was performed (1:500;) in 0.8% BSA/PBS (Sigma-Aldrich, Steinheim, Germany) for 60 minutes at room temperature and nuclei were stained with 2 μg/ml Hoechst 33342 (Life Technologies). Images were acquired using the high content screening (HCS) imaging system Operetta® and Harmony® software (PerkinElmer, Waltham, MA, USA).

### Electron microscopy

Cells were fixed by immersion with 3 to 6% (v/v) glutaraldehyde in 0.1 M sodium cacodylate buffer, rinsed twice for 15 minutes in 0.1 M cacodylate buffer, pH 7.2, post-fixed for 120 minutes with 1% OsO_4_ in 0.1 M cacodylate buffer, pH 7.2, at room temperature in the dark, rinsed again, dehydrated with acetone and embedded in araldite CY212 (Durcupan ACM, Fluka, Sigma, St.Louis, MO, USA). Ultrathin sections were cut at grey interference colour (25 to 30 nm) with a 35°-diamond knife (Diatome, Hatfield, PA, USA) on an Ultracut E (Leica, Buffalo Grove, IL, USA), mounted on formvar/carbon-coated copper grids and contrasted with uranyl acetate and lead citrate. Microscopy was performed with a Zeiss EM109 (80 kV, 500 μm condenser 1 aperture, 200 μm condenser 2 aperture, 30um objective aperture; Carl Zeis Jena, Germany) equipped with a temperature-stabilized wide angle YAC-CCD camera at the side entry port (1024 × 1024 pixel, 12-bit greyscale/pixel; info@trs-system.de). Magnification was calibrated with a cross grating replica (2,160 lines/mm, d = 0.463 μm).

### Recording of intracellular calcium transients

iPS-CMs cultured in chamber slides were loaded with 2 μM Fura 2-AM (Molecular Probes, Life Technologies, Carlsbad, CA, USA) in Hank’s balanced salts modified buffer (HBSS, pH 7.4) for 15 minutes at 37°C, and washed twice for 15 minutes in HBSS. The slides were placed on a temperature-regulated microscope stage and kept at 37°C. Fluorescence images were acquired using the IonWizard Transient Analysis software package (IonOptix Limited, Dublin, Ireland) together with an Olympus IX70 inverted fluorescence microscope (40× Fura-UV objective) (Myocyte Calcium & Contractility Recording System; Ion Optix Limited). The data shown represent the average of Fura-2 ratio (R340/380) for 35 iPS-CMs from three independent experiments.

### Determination of oxygen consumption rate

For oxygen consumption rate (OCR) quantification, 20,000 iPS-CMs were seeded into each well of a Seahorse XFe96 cell culture plate and cultured for 2 days to ensure firm adherence to the cell culture plate. On the day of measurement, the medium was changed to pre-warmed Seahorse Assay medium and OCR determined according to the manufacturer’s instructions using the Seahorse XF Cell Mito Stress Kit (all from Seahorse Bioscience, North Billerica, MA, USA). Basal respiration was measured in unstimulated cells during three cycles of measurement. Afterwards, oligomycin (1 μM) was added to quantify ATP production followed by carbonyl cyanide-4-(trifluoromethoxy)phenylhydrazone (FCCP; 1 μM) injection to assess maximal cellular respiration (respiratory capacity). Finally, antimycin A and rotenone (1 μM) treatment revealed the amount of non-mitochondrial respiration.

### Electrophysiology

iPS-CMs and N-CMs aggregates were dispersed using collagenase B [17] or trypsin/EDTA, respectively, followed by addition of neutralization solution (all Provitro). Single cells were incubated at 37°C in DMEM/20% metal calf serum (FCS) for 24 to 36 hours before measurements were performed. Action potentials (APs) of spontaneously beating CMs were measured by means of the whole-cell current clamp technique using an EPC-9 amplifier and the PULSE software package (Heka Elektronik, Lambrecht, Germany). The experiments were performed at 37°C in standard extracellular solution containing (in mMol/l) 140 NaCl, 5.4 KCl, 1.8 CaCl_2_, 1 MgCl_2_, 10 HEPES and 10 glucose, or 136 NaCl, 5.4 KCl, 0.33 NaH_2_PO_4_, 1 MgCl_2_, 10 glucose, 5 HEPES and 1.8 CaCl_2_ (pH 7.4, adjusted with NaOH). Patch-clamp pipettes, prepared from glass capillary tubes (Harvard Apparatus Ltd, Kent, UK) with a two-stage horizontal puller (DMZ Universal Puller, Munich, Germany), had a resistance of 2 to 3 MΩ when filled with intracellular solution containing (in mMol/l) 50 KCl, 80 KAspartate, 1 MgCl_2_, 3 MgATP, 10 EGTA and 10 HEPES (pH 7.4).

Isoproterenol and carbachol (Sigma-Aldrich, Steinheim, Germany) were prepared freshly before the experiments and applied using gravitational flow. Data were analyzed off-line with custom-made analysis software (kindly provided by Prof. Philipp Sasse, University of Bonn, Germany).

### Exposure of cells to hypoxia and glucose/serum deprivation

Puromycin-selected iPSC-derived cardiac clusters were dissociated at day 16 of differentiation with 0.25% trypsin-EDTA and plated on a 2.5 μg/ml fibronectin-coated 96-well plate at the density of 40,000 cells/well in high-glucose DMEM, supplemented with 15% FBS, 1% non-essential amino acids, and 50 μM β-mercaptoethanol (all from Life Technologies). Puromycin (8 μg/ml) was maintained in culture medium and refreshed every second day to prevent the outgrowth of any proliferating cells that may contaminate purified CM preparations. Five days after plating and culture in a humidified incubator (CB 210; Binder, Tuttlingen, Germany) at 37°C, 5% CO_2_ and atmospheric oxygen concentration (21%), parallel plates were transferred to an incubator (CB 150; Binder) maintained at 1% O_2_ and incubated for 3, 5 or 7 hours in: a) basal DMEM, high glucose supplemented with both 10% FBS and glucose; b) DMEM, no glucose containing only 10% FBS; c) DMEM, high glucose containing only glucose; or d) DMEM, no glucose lacking both serum and glucose. Control cells were kept for equivalent time periods under normoxic standard conditions. After establishing the conditions leading to reproducibly detectable cell damage, all subsequent experiments were performed by incubating iPS-CMs and N-CMs for 3 hours in hypoxia (1% O_2_) in glucose- and serum-deprived medium as summarized in Figure 1A. The analyses of various cell viability parameters were performed at the end of the hypoxia period, without reoxygenation. The number and density of iPS-CMs and N-CMs incubated under control conditions was similar as determined by cell counting at the end of the incubation period, using the Operetta® HCS.

### Caspase activity

For detection of cells undergoing apoptosis, iPS-CMs and N-CMs were plated in 96-well ViewPlates (PerkinElmer). Poly-caspase-active cells were visualized using the fluorescent poly-caspase reagent, R-VAD-FMK (SR-FLICA; Biomol, Hamburg, Germany), a sensitive and early detector of apoptosis initiation. Following incubation under control conditions or hypoxia and/or GSD, medium was aspirated, 60 μl/well of SR-FLICA reagent was added, and cells were incubated for the next 45 minutes at 21% O_2_. Cells were washed with Dulbecco’s PBS, fixed with 4% PFA, and nuclei were stained with 2 μg/ml Hoechst 33342 (Life Technologies). Finally, cells were visualized and quantitatively analyzed using the Operetta® HCS. In addition, the activity of caspase-3/7, -8 and -9 was assessed using specific substrates reconstituted in respective caspase-Glo-3/7, -8 or -9 buffer (Promega, Mannheim, Germany). Following incubation in hypoxia/GSD and control conditions, 60 μl of reagent was added to an equal volume of medium in 96-well transparent plates (Greiner Bio-One, Frickenhausen, Germany), and incubated for 1 hour at room temperature; 100 μl from each well was then transferred to opaque 96-well plates and luminescence, directly proportional to the amount of caspase activity, was recorded using a Mithras LB 940 Multimode Microplate Reader (Berthold Technologies, Bad Wildbad, Germany).

### 7-aminoactinomycin D staining

Late apoptotic and necrotic cells were detected using 7-aminoactinomycin D (7-AAD) (Biomol). 7-AAD was reconstituted in 260 μl DMSO and diluted 1:250 in DMEM (high glucose) supplemented with 10% FBS. Following incubation in hypoxia or control conditions, 60 μl of reagent was added to cells, which were then incubated for 15 minutes at room temperature. Cells were fixed with 4% PFA and nuclei stained with Hoechst 33342. Quantitation of 7-AAD-positive cells was performed using the Operetta® HCS.

### TUNEL staining

DNA fragmentation was detected using the *in situ* cell death detection kit (Roche, Mannheim, Germany). Cells were fixed with 4% PFA for 60 minutes at room temperature and permeabilized with 0.25% Triton X-100. After washing in PBS, reagent was added for 60 minutes at 37°C followed by Hoechst 33342 staining. Cells were visualized and quantitatively analyzed using the Operetta® HCS.

### Mitochondrial membrane integrity

Analysis of the mitochondrial membrane potential was performed in control cells and cells subjected to hypoxia/GSD using the JC-1 Mitochondrial Membrane Potential Probe (Life Technologies). JC-1 was added into medium at 2 μM final concentration for 30 minutes incubation at room temperature. Then, cells were fixed with 4% PFA and nuclei stained with Hoechst 33342. The loss of “granulated” JC-1 staining in CMs and conversion into a homogenous staining pattern indicated breakdown of the mitochondrial membrane potential [18]. Quantification was performed using the Operetta® HCS.

### Mitochondrial transmembrane potential

The mitochondrial transmembrane potential was measured using the tetramethylrhodamine methyl ester (TMRM) fluorescence method (Life Technologies). TMRM dye was added to the medium at 125 nM final concentration for 30 minutes incubation at 37°C, followed by 3 minutes incubation with Hoechst 33342. The loss of mitochondrial membrane potential causes TMRM to leak from mitochondria, resulting in a loss of fluorescence intensity. Live images of cells were recorded for TMRM fluorescence and quantitation of fluorescence intensity was performed using the Operetta® HCS.

### Reactive oxygen species measurement

Oxidative stress induced by reactive oxygen species (ROS) was measured using CellROX® Deep Red Reagent (Life Technologies). CellROX® reagent was added at a final concentration of 5 μM to the cells and incubated for 30 minutes at 37°C. After washing in PBS, cells were fixed with 4% PFA and nuclei stained with Hoechst 33342. Images were acquired and quantification of the fluorescence signal was performed using the Operetta® HCS.

### MTS assay

Metabolic activity was analyzed using the colorimetric CellTiter 96 AQueous Non-Radioactive Cell Proliferation Assay (Promega), based on the reduction of tetrazolium dye MTT (3-(4,5-dimethylthiazol-2-yl)-2,5-diphenyltetrazolium bromide) to its insoluble formazan by NADPH-dependent oxidoreductases. Following 3 hours incubation (40,000 CMs/well) in 1% O_2_ in hypoxia/GSD or control conditions, 70 μl of the MTS/PMS solution was added at 50 μl medium/well and incubated for 4 hours at 21% O_2._ Absorbance was measured at 490 nm directly in the 96-well plate using a SpectraMax Plus384 Absorbance Microplate Reader (Molecular Devices, Sunnyvale, CA, USA). The number of cells/well was counted using the Operetta® HCS, and was confirmed to be similar between experimental groups. Blank values from wells without cells were subtracted.

### ATP quantification

Cardiomyocyte ATP content was analyzed using the CellTiter-Glo Luminescent Cell Viability Assay (Promega). Briefly, 60 μl reagent (CellTiter-Glo buffer/substrate) was added (1:1) to 40,000 CMs/well cultured in 96-well plates following incubation in hypoxia/GSD or control conditions, and the plate was placed on an orbital shaker at room temperature for 2 minutes (200 rpm). The content of the plate was left to equilibrate for an additional 10 minutes at room temperature, before 100 μl from each well were transferred to opaque 96-well plates and luminescence was recorded using a Mithras LB 940 Multimode Microplate Reader (Berthold Technologies). The number of cells/well was counted using the Operetta® HCS and was confirmed to be similar between experimental groups. Blank values from cell-free wells filled with medium and CellTiter-Glo buffer/substrate were substracted.

### Preparation of mesenchymal stromal cell-conditioned medium and fibroblast-conditioned medium

Bone marrow-derived MSCs were isolated from 8-week-old C57BL/6 mice and characterized as previously described [19]. The local Animal Ethics Committee (Deutsches Herzzentrum and Charite-Universitätsmedizin Berlin) granted approval, and all procedures conformed to the guidelines from Directive 2010/63/EU of the European Parliament. Fibroblasts were isolated from CF-1 mouse strain at E13.5-E14.5 according to standard protocols. Fibroblasts and MSCs were grown until 80 to 90% confluence in IMDM medium (PAN Biotech, Aidenbach, Germany) supplemented with 5% FBS and 2.5 ng/ml recombinant human basic fibroblast growth factor (Peprotech, Hamburg, Germany) in 21% O_2_. For the collection of conditioned medium, cells were washed thoroughly twice with Dulbecco's PBS and cultured in serum/glucose-deprived medium at 1% O_2_ for 72 hours. After 72 hours, conditioned medium was collected and centrifuged for 5 minutes at 300 x g at room temperature. Equivalent medium kept under the same conditions in culture flasks without cells was used as a control. Cell viability after 72 hours exposure to hypoxia/GSD was evaluated by Trypan blue exclusion.

To determine the effect of MSC-conditioned medium (MSC-CoM) and fibroblast-conditioned medium (FB-CoM) on the viability of iPS-CMs exposed to hypoxia and nutrient deprivation, iPS-CMs and N-CMs cells maintained under baseline conditions (DMEM in 15% FBS and glucose, 21% O_2_) were washed twice with Dulbecco's PBS, and standard culture medium was replaced with DMEM without FBS and without glucose or with MSC-CoM/FB-CoM (60 μl/well in a 96-well plate) for 3 hours incubation at 1% O_2_. Cell viability parameters were analyzed immediately after the experiment.

### RNA extraction, cDNA synthesis and real-time PCR

Total RNA was extracted using the RNeasy Mini Kit (Qiagen, Hilden, Germany); 5 μg RNA per sample were separated on 1% agarose gels to confirm RNA integrity, and RNA concentration and quality were assessed by spectrophotometry. First-strand cDNA was synthesized from 1 μg total RNA using Oligo (dT) primers and Superscript™ II RNase Reverse Transcriptase (Life Technologies). Quantification of selected genes by real-time PCR was performed in triplicate using the Mastercycler® ep realplex (Eppendorf, Hamburg, Germany) with SYBR Green and PCR Master Mix (Life Technologies). Each reaction consisted of 1 μl cDNA, 2.5 μl primer (5 μM) and 21.5 μl reaction buffer (Platinum SYBR Green; Life Technologies) in a total reaction volume of 25 μl. Real-time PCR cycles consisted of: 10 minutes at 95°C for polymerase activation, 40 cycles of 15 seconds at 95°C, 15 seconds at 60 to 68°C, 45 seconds at 68°C; and for the *Gata4*, *Mef2c*, *Myh6*, *Nkx 2.5*, *Tnnt2*: 10 minutes at 95°C for polymerase activation, 40 cycles of 15 seconds at 95°C and 60°C for 1 minutes. GAPDH was amplified to serve as an intrinsic control. The data were analyzed by the Pfaffl method [20]. The details of the primers used are given in Table 1.

**Table 1:** **Primers used for real-time quantitative PCR**

**Gene**	**Primer sequence (5′→3′)**	**Annealing temperature (°C)**	**Reference**
*Hif 1*α	Fw: CATGATGGCTCCCTTTTTCA	63	[55]
	Rev: GTCACCTGGTTGCTGCAATA		
*Hif 2*α	Fw: GGGAACACTACACCCAGTGC	63	[55]
	Rev: TCTTCAAGGGATTCTCCAAGG		
Vegf	Fw: ACTGGACCCTGGCTTTACTG	63	[56]
	Rev: TCACTTCATGGGACTTCTGC		
*Glut-1*	Fw: GCTTCCTGCTCATCAATCGT	63	[56]
	Rev: CTTCTTCTCCCGCATCATCT		
*HSP70*	Fw: TGGTGCTGACGAAGATGAAG	63	[57]
	Rev: AGGTCGAAGATGAGCACGTT		
*PKC*δ	Fw: CAGACCAAGGACCACCTGTT	63	[58]
	Rev: GCATAAAACGTAGCCCGGTA		
*PKC*ε	Fw: GAGGACTGGATTGACCTGGA	63	[58]
	Rev: ATCTCTGCAGTGGGAGCAGT		
*PKC*θ	Fw: ATGGACAACCCCTTCTACCC	63	[58]
	Rev: GCGGATGTCTCCTCTCACTC		
*Bcl2*	Fw: TGTGTGTGGAGAGCGTCAACA	65	[59]
	Rev: TGCCGGTTCAGGTACTCAGTC		
*Bax*	Fw: GCGTGGTTGCCCTCTTCTACTTTG	65	[60]
	Rev: AGTCCAGTGTCCAGCCCATGATG		
*Bad*	Fw: GGGAGCAACATTCATCAGCAGG	65	[60]
	Rev: CATCCCTTCATCCTCCTCGGTC		
*Bcl-xL*	Fw: AACATCCCAGCTTCACATAACCCC	65	[60]
	Rev: GCGACCCCAGTTTACTCCATCC		
*P53*	Fw: ATTTGTATCCCGAGTATCTG	63	[56]
	Rev: GGTATACTCAGAGCCGGCCT		
*Fas*	Fw: AGGACTGCAAAATGAATGGG	60	[61]
	Rev: GGGTGCAGTTTGTTTCCACT		
*FADD*	Fw: GGGGACTCATCCTGTTTTCT	60	[62]
	Rev: ATGCATAGTCTGGGGAGTCA		
*Gata4*	Fw: AATGCCTGTGGCCTCTATCA	60	[63]
	Rev: GGTCTCGCTCCTGGAAGATG		
*Mef2c*	Fw: ATTTGGGAACTGAGCTGTGC	60	[63]
	Rev: CGCTCATCCATTATCCTCGT		
*Myh6*	Fw: CCAACACCAACCTGTCCAAGT	60	[64]
	Rev: AGAGGTTATTCCTCGTCGTGCAT		
*Nkx2.5*	Fw: CCAGAACCGTCGCTACAAGT	60	[65]
	Rev: GGGTAGGCGTTGTAGCCATA		
*Tnnt2*	Fw: GCCAAAGATGCTGAAGAAGG	60	[66]
	Rev: TTCTCGAAGTGAGCCTCGAT		
*Gapdh*	Fw: GGGGACTCATCCTGTTTTCT	60	[64]
	Rev: ATGCATAGTCTGGGGAGTCA		

Fw, forward; Rev, reverse.

### Western blot

Murine iPS-CMs cells were lysed in SDS lysis buffer supplemented with complete proteinase inhibitor cocktail and PhosSTOP phosphatase inhibitor cocktail tablets (Roche Diagnostics, Mannheim, Germany). Protein concentration was determined by BCA protein assay (Thermo Scientific, Bonn, Germany). Aliquots containing equal amounts of proteins (20 to 30 μg) were resolved by 10% SDS-PAGE and transferred onto nitrocellulose membranes (Karl Roth, Karlsruhe, Germany). Membranes were blocked and incubated with monoclonal mouse anti-total-protein and rabbit anti-phospho-protein primary antibodies overnight at 4°C: PKCε (1:500, cat. no. sc-214, Santa Cruz Biotechnology, Dallas, Texas, USA), Akt (1:2000, cat. no. 2920, Cell Signaling, Danvers, Massachusetts, USA), phospho-Akt (Ser473, 1:2000, cat. no. 4060, Cell Signaling), STAT3 (1:1000, cat. no. 9139, Cell Signaling), and phospho-STAT3 (Tyr705, 1:2000, cat. no. 9145, Cell Signaling). GAPDH served as a loading control (1:1000, cat. no. 2118, Cell signaling). After 1 hour incubation with IRDye® conjugated secondary antibodies (Li-Cor Bioscience, Bad Homburg, Germany), blots were analyzed using the infrared imaging system and software Odyssey® from Li-Cor Bioscience and quantified by ImageJ software.

### Statistical analysis

For every analysis, at least three independent experiments were performed, each done in triplicate. Data are presented as mean value ± SEM. Data from two independent groups of samples were compared using Student’s *t* test. Three or more groups of samples were compared using one-way analysis of variance with Bonferroni *post-hoc* analysis. A *P* value ≤0.05 was considered statistically significant. Tests were carried out using IBM SPSS Statistics 20 software (IBM, Armonk, NY, USA).

## Results

### Induced pluripotent stem cell-derived cardiomyocyte differentiation

Spontaneously beating, GFP-positive areas in EBs were first observed on days 8 to 9 of differentiation (Figure 1B). After puromycin selection, the remaining cell clusters were composed exclusively of GFP-positive cells, which represented more than 95% of all viable cells as determined by flow cytometry of enzymatically dissociated clusters at day 16 (Figure 1C). When these cells were plated and cultured for an additional 5 days on fibronectin-coated plates in the presence of puromycin, they all stained positive for cardiac sarcomeric protein α-actinin (Figure 1D).

### Induced pluripotent stem cell-derived cardiomyocyte phenotype and function

**Figure 2:**
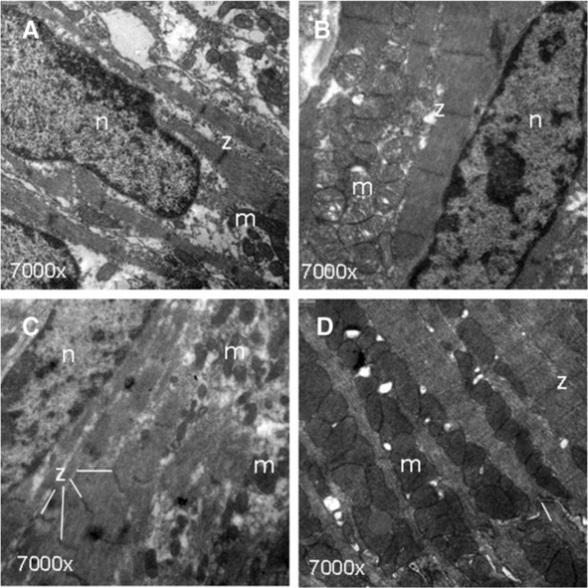
Transmission electron microscopy. **(A)** Murine late embryonic (E16.5), **(B)** neonatal, and **(C)** induced pluripotent stem cell-derived cardiomyocytes (iPS-CMs; day 18 of differentiation). For comparison, **(D)** shows cardiomyocytes in an adult mouse heart. Myofibrils with typical cross striations surrounded by mitochondria are present in all cells but are densely packed and organized only in adult cardiomyocytes. The morphology of iPS-CMs is similar to that of late embryonic/early neonatal cardiomyocytes. m, Mitochondria; n, nucleus; z, sarcomeric z-line.

**Figure 3:**
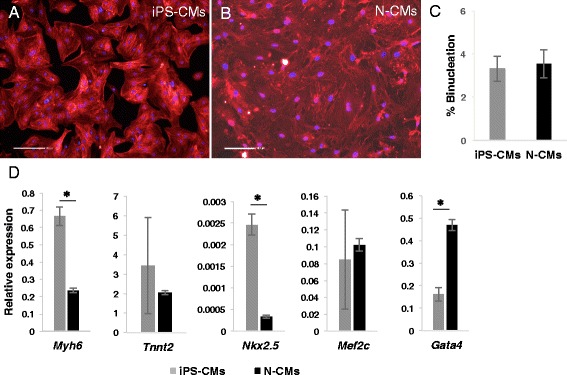
Phenotypic features of induced pluripotent stem cell-derived cardiomyocytes (iPS-CMs) and neonatal cardiomyocytes (N-CMs). **(A,B)** Sarcomeric alpha-actinin staining of iPS-CMs and N-CMs 5 days after plating taken at individually adjusted exposure intensity. Nuclei are DAPI stained, scale bar 100 μm. **(C)** Percentage of binucleated cells. **(D)** Expression of selected genes relevant for cardiomyocyte development determined by real-time PCR. *P < 0.05.

**Figure 4:**
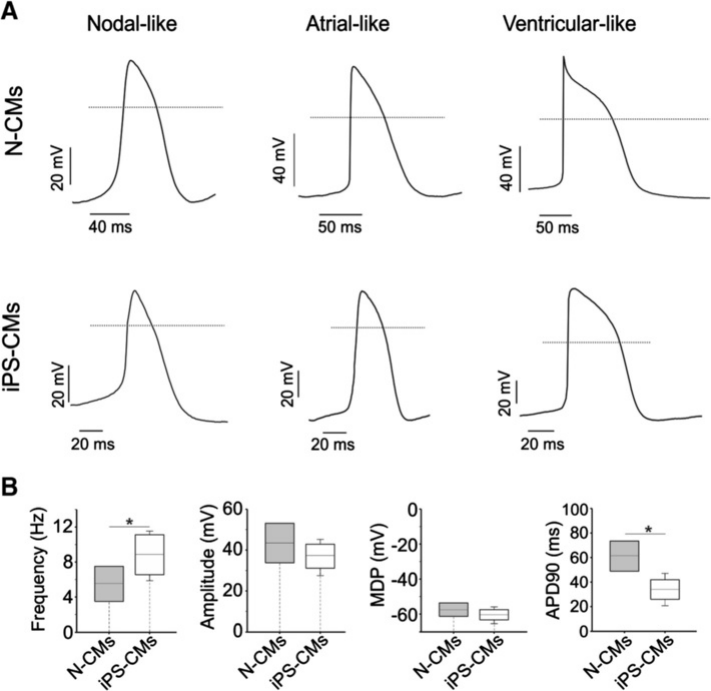
Electrophysiology properties of neonatal cardiomyocytes (N-CMs) and induced pluripotent stem cell-derived cardiomyocytes (iPS-CMs). **(A)** Current clamp recordings revealed differentiation of neonatal cells into atrial and ventricle-like action potentials from cardiomyocytes. **(B)** Comparison of action potential parameters measured from N-CMs and iPS-CMs. Solid lines through distributions indicate population means. Dotted lines indicate 0 mV membrane potential (A) or population means (B), respectively. Data were compared using *t*-test. *P < 0.05 between both cell types. MDP, maximum diastolic potential; APD90, action potential duration at 90% of repolarization.

**Figure 5:**
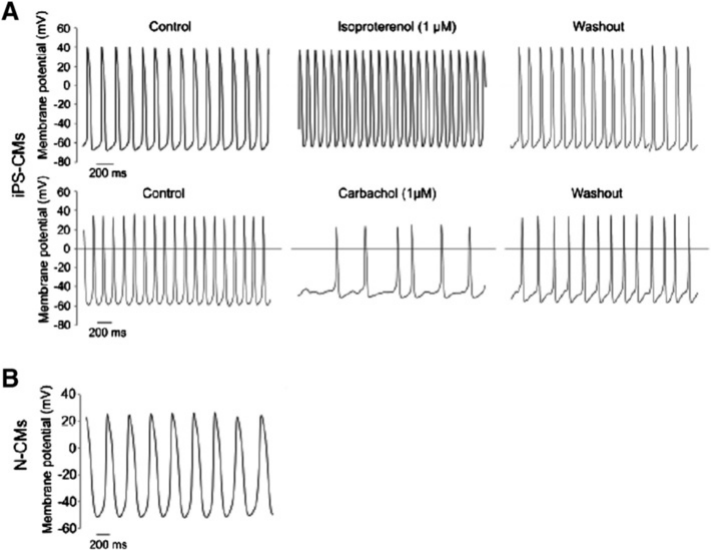
Functional characteristics of induced pluripotent stem cell-derived cardiomyocytes (iPS-CMs) and neonatal cardiomyocytes (N-CMs). **(A)** Representative action potential (AP) recording traces of iPS-CMs showing the positive and negative chronotropic response to isoproterenol and carbachol application, respectively. **(B)** AP recordings from spontaneously beating N-CMs.

**Figure 6:**
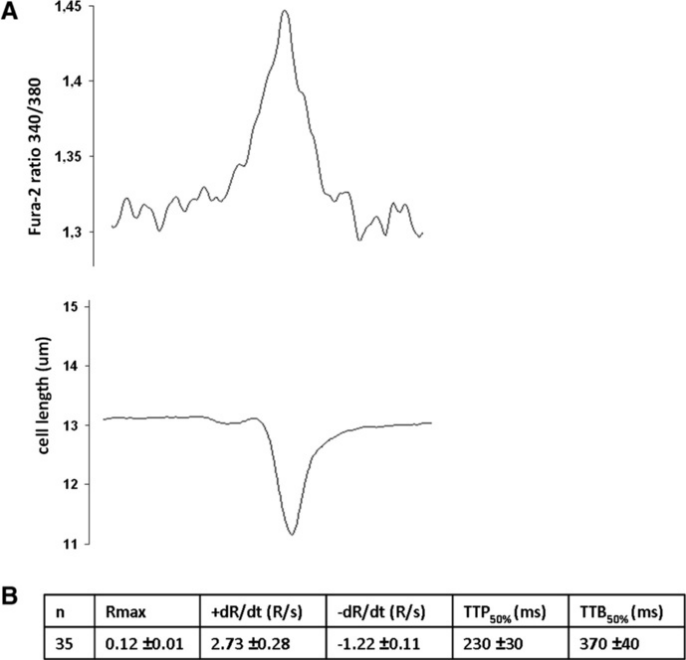
Calcium transient analysis in induced pluripotent stem cell-derived cardiomyocytes. **(A)** Representative recordings of Fura-2-AM fluorescence ratio at 340/380 nm excitation wavelengths and cell shortening. **(B)** Quantitative analysis of intracellullar calcium transient dynamics. R is the Fura 2-ratio at 340/360 nm. R_max_, maximal systolic calcium amplitude (the difference of R-systolic and R-diastolic); +dR/dt, maximal rate of fluorescence rise during sytole; −dR/dt, maximal rate of fluorescence decay during diastole; TTP50%, time to 50% peak (in ms); TTB50%, time to 50% baseline (ms). Data are means ± SEM, N = 35; cells studied in three independent experiments.

**Figure 7:**
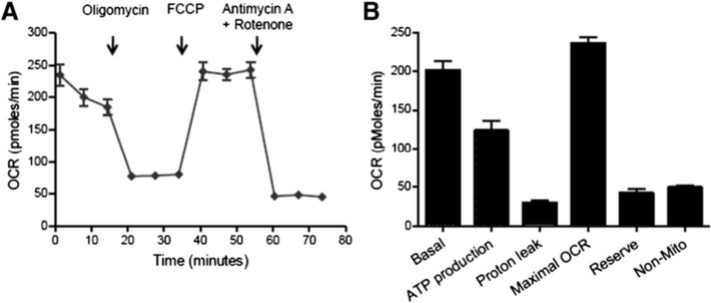
Analysis of mitochondrial respiration of induced pluripotent stem cell-derived cardiomyocytes using the Seahorse XF Cell Mito Stress Kit. **(A)** Oxygen consumption rate (OCR) under basal and under different conditions of mitochondrial stress that was induced by addition of substances indicated on top of the panel. Three cycles of measurements were performed for each condition. **(B)** Individual mitochondrial function parameters calculated from the data shown in (A). Data in (A) and (B) are shown as mean ± SEM for n = 7 replicates. FCCP, carbonyl cyanide 4-(trifluoromethoxy)phenylhydrazone; non-Mito, non-mitochondrial oxygen consumption.

As demonstrated by electron microscopy (Figure 2), the ultrastructure of iPS-CMs mirrored that of late embryonic and neonatal naïve CMs, and the maturation process had clearly not reached the level of adult CMs. By light microscopy, cell size was similar between iPS-CMs and N-CMs, and the small percentage of binucleated cells confirmed the relative immaturity of both cell types (Figure 3A-C). The mRNA expression pattern of selected genes that are relevant to CM development was heterogenous (Figure 3D). Of the earlier transcription factors, *Nkx2.5* was higher in iPS-CMs than in N-CMs, *Gata4* was lower and *Mef2c* similar. Among genes encoding for mature contractile proteins, *Myh6* was higher in iPS-CMs and *Tnnt2* was not significantly different. Overall, expression level variability (that is, standard error) was higher in iPS-CMs, indicating the heterogeneity and ongoing maturation within the reprogrammed cell population. Current clamp recordings indicated that both iPS-CMs and N-CMs displayed ventricular-, atrial-, and nodal-like AP morphologies (Figure 4A). Both iPS-CMs and N-CMs showed similar maximum diastolic potential and amplitude (Figure 4B). However, the beating frequency and AP duration at 90% of repolarization (APD90) were significantly different, with the frequency being lower (5.10 ± 2.47 Hz) and the APD90 longer (60.53 ± 8.30 ms) in N-CMs than in iPS-CMs (8.41 ± 2.44 Hz and 34.19 ± 8.33 ms; both *P* > 0.05; Figure 4B). iPS-CMs responded to the adrenergic agonist isoproterenol with increased beating frequency, whereas the muscarinergic agonist charbachol exerted a negative chronotropic effect (Figure 5A). Both effects could be reversed upon washout. Overall, AP tracings of N-CMs were comparable (Figure 5B), but the response to pharmacological agents could not be determined due to cell instability shortly after gigaseal formation (data not shown). iPS-CMs also displayed intact excitation-contraction coupling as indicated by simultaneous recordings of intracellular calcium transients (Fura 2-AM fluorescence ratio (R_340/380_)) and cellular contraction/shortening, but the slow upstroke of the calcium transients confirmed their relative immaturity (Figure 6A). The quantitative data on calcium transient morphology and dynamics, averaged from 35 individual recordings, are given in Figure 6B and confirm the fetal/neonatal character of the iPS-CMs. In line with this notion, the basal respiration rate in iPS-CMs was 201 ± 35 pmoles O_2_/minute (Figure 7). After three basal measurements, the ATP synthase inhibitor oligomycin was added to differentiate the ATP-linked respiration (123 ± 35 pmoles/minute) from the proton leak (29 ± 12 pmoles/minute). The addition of the uncoupling agent FCCP increased the OCR by iPS-CMs to 236 ± 21 pmoles/minute (maximal respiration) and revealed a residual respiratory capacity of 35 ± 21 pmol/minute. The rate of oxygen consumption due to non-mitochondrial sources was assessed by addition of complex I and III inhibitors rotenone and antimycin A, respectively, and shown to be 49 ± 11 pmol/minute.

### Induced pluripotent stem cell-derived cardiomyocyte hypoxia and glucose/serum deprivation *in vitro* model

**Figure 8:**
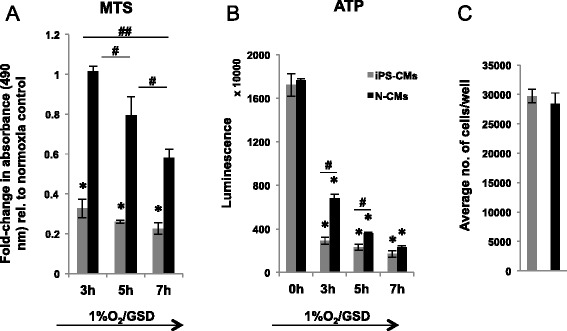
Effect of hypoxia and nutrient deprivation on metabolic activity of induced pluripotent stem cell-derived cardiomyocytes (iPS-CMs) and neonatal cardiomyocytes (N-CMs). **(A)** Activity of cellular oxidoreductases in iPS-CMs and N-CMs determined by MTS assay, normalized to normoxia/full medium data. *P ≤ 0.05, for iPS-CMs versus normoxia (Bonferroni test); ^#^ *P* ≤ 0.05, versus different hypoxia and glucose/serum deprivation (GSD) durations (Bonferroni test); ^##^ *P* < 0.001, iPS-CMs versus N-CMs by analysis of variance. **(B)** ATP content in iPS-CMs and N-CMs determined by CellTiter Glo assay. *P ≤ 0.05, versus normoxia (0 hours, Bonferroni test); ^#^ *P* ≤ 0.05, versus N-CMs. **(C)** Average number of iPS-CMs and N-CMs per well.

In the presence of serum and glucose, hypoxia alone for up to 7 hours did not lead to a markedly increased proportion of poly-caspase active iPS-CMs compared to cells incubated in full medium (15% FBS and high glucose) at 21% O_2_ (Figure 1E). Similarly, hypoxia and concomitant withdrawal of either serum or glucose alone did not significantly influence the number of poly-caspase-positive cells. However, iPS-CMs cultured at 1% O_2_ in serum- and glucose-deprived medium displayed a relevant time-dependent increase in the number of poly-caspase active cells, and 48 ± 3% were poly-caspase-positive after 3 hours incubation in combined hypoxia and GSD (*P* = 0.001 versus baseline). After 5 hours, 67 ± 2% (*P* = 0.006 versus 3 hours) and, after 7 hours, 96 ± 2% of the iPS-CM were stained positive (*P* = 0.0003 versus 5 hours; Figure 1E). Similarly, the MTS conversion rate dropped by 67 ± 3.3% after 3 hours, 73 ± 0.5% after 5 hours, and 77 ± 2% after 7 hours hypoxia/GSD (Figure 8A). We therefore chose 3 hours hypoxia and GSD as the model for all subsequent experiments, as it leaves sufficient room for the detection of both an improvement and worsening of the outcome.

### Neonatal cardiomyocyte and induced pluripotent stem cell-derived cardiomyocyte response to hypoxia and glucose/serum deprivation

There were distinct differences between iPS-CMs and N-CMs regarding the preservation of metabolic activity in hypoxia/GSD. Cytosolic and mitochondrial oxidoreductase activity was reduced by 67 ± 3.3% in iPS-CMs, but did not change in N-CMs after 3 hours hypoxia/GSD (−0.014 ± 1.8%; *P* < 0.001; Figure 8A). We therefore followed oxidoreductase activity for longer periods of hypoxia GSD and found that it dropped by 73 ± 0.5% in iPS-CMs and by 20 ± 6.5% in N-CMs after 5 hours (*P* < 0.001), and by 77 ± 2% in iPS-CMs and 42 ± 3.1% in N-CMs after 7 hours (*P* < 0.001), confirming a higher sensitivity to hypoxia/GSD of iPS-CMs throughout the experimental protocols (Figure 8A). Furthermore, we assessed cytoplasmic ATP levels, which were similar in iPS-CMs and N-CMs in normoxic control conditions (Figure 8B). During 3 hours hypoxia/GSD, however, cytoplasmic ATP decreased by 83 ± 0.9% in iPS-CMs and by 61 ± 0.4% in N-CMs (*P* < 0.001).

**Figure 9:**
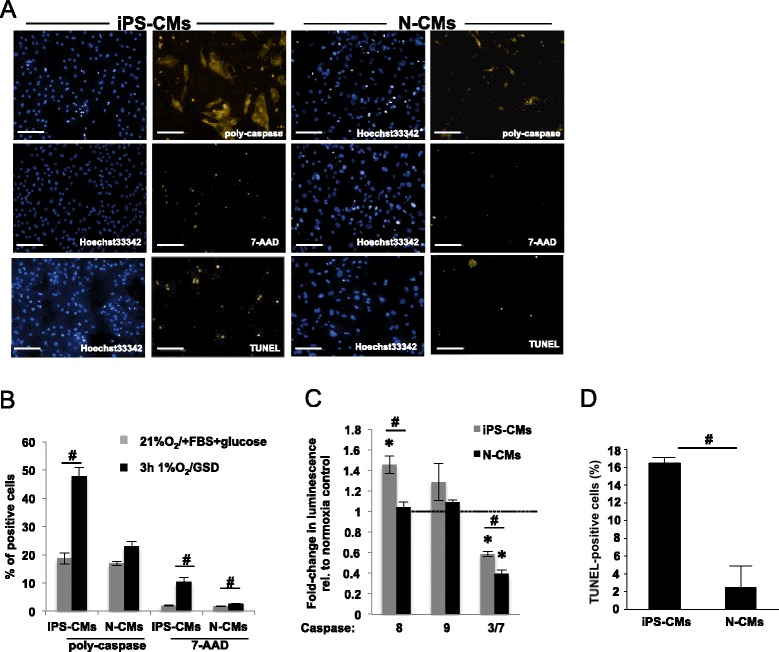
Response of murine induced pluripotent stem cell-derived cardiomyocytes (iPS-CMs) and neonatal cardiomyocytes (N-CMs) to *in vitro* hypoxia and nutrient deprivation. **(A)** Representative photomicrographs (scale bar 100 μm) and **(B)** quantitative analysis of poly-caspase-positive, 7-aminoactinomycin D (7-AAD)-positive iPS-CMs and N-CMs after 3 hours incubation in hypoxia and glucose/serum deprivation (GSD) (^#^ *P* ≤ 0.05, versus normoxia/full medium). **(C)** Caspase-8, -9, and 3/7 activities in iPS-CMs and N-CMs after 3 hours incubation in hypoxia/GSD. Data are normalized to those normoxia controls. *P ≤ 0.05, versus normoxia/full medium; ^#^ *P* ≤ 0.05, iPS-CMs versus N-CMs in hypoxia/GSD. **(D)** Increase in TUNEL-positive cells after 3 hours incubation in hypoxia/GSD (^#^ *P* = 0.001). FBS, fetal bovine serum.

**Figure 10:**
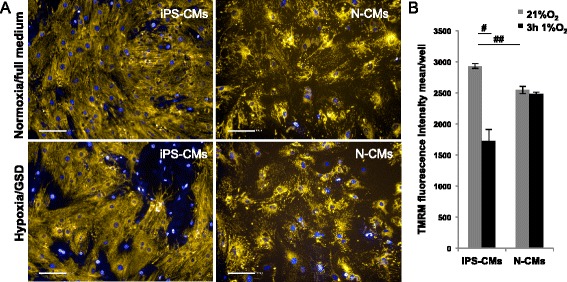
Mitochondrial potential analysis in induced pluripotent stem cell-derived cardiomyocytes (iPS-CMs) and neonatal cardiomyocytes (N-CMs). **(A)** Representative images of tetramethylrhodamine methyl ester (TMRM) staining of iPS-CMs and N-CMs after 3 hours hypoxia and glucose/serum deprivation (GSD) compared to normoxia control (21% O_2_, 15% fetal bovine serum and high glucose). Scale bar 100 μm. **(B)** Quantification of TMRM fluorescence intensity of cells cultured in normoxia versus cells cultured in hypoxia and GSD (^#^ *P* < 0.001, versus normoxia control; ^##^ *P* < 0.001, iPS-CMs versus N-CMs in normoxia).

**Figure 11:**
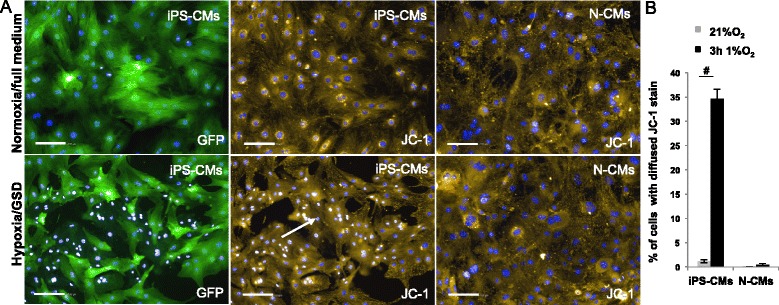
Mitochondrial potential analysis in induced pluripotent stem cell-derived cardiomyocytes (iPS-CMs) and neonatal cardiomyocytes (N-CMs). **(A)** Representative images of JC-1 staining of iPS-CMs and N-CMs after 3 hours hypoxia and glucose/serum deprivation (GSD) compared to normoxia control (21% O_2_, 15% fetal bovine serum and high glucose) (^#^ *P* < 0.01, versus normoxia control). Scale bar 100 μm. Note the nuclear shrinking in cells with diffuse JC-1 staining. **(B)** Proportion of cells with diffuse JC-1 staining expressed as % of cells versus normoxia/full medium (^#^ *P* ≤ 0.01). GFP, green fluorescent protein; JC-1, 5,5′,6,6′-tetrachloro-1,1′,3,3′-tetraethylbenzimidazol-carbocyanine iodide.

**Figure 12:**
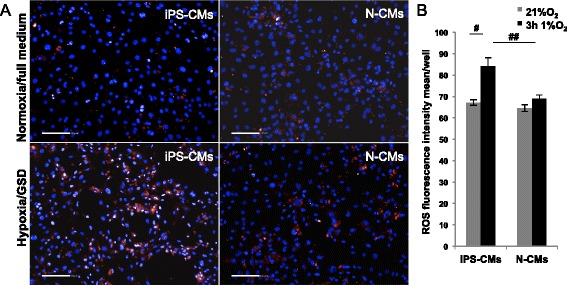
Reactive oxygen species (ROS) expression analysis. **(A)** Representative images of ROS accumulation in cells with exacerbated oxidative stress, visualized using the CellROX assay®. In induced pluripotent stem cell-derived cardiomyocytes (iPS-CMs) and neonatal cardiomyocytes (N-CMs) after 3 hours hypoxia and glucose/serum deprivation (GSD) compared to normoxia control (21% O_2_, 15% fetal bovine serum and high glucose). Scale bar 100 μm. **(B)** Quantification of oxidative stress (CellROX® fluorescence intensity) in iPS-CMs and N-CMs cultured in normoxia versus those cultured in hypoxia and GSD (^#^ *P* = 0.005, versus normoxia control; ^##^ *P* = 0.002, iPS-CMs versus N-CMs in hypoxia).

In contrast to iPS-CMs, which reacted to 3 hours hypoxia and GSD by a 48 ± 3% increase of poly-caspase active cells, only 23 ± 2% of the N-CMs displayed poly-caspase activity after exposure to the same conditions (*P* = 0.002; Figure 9). Regarding the differential activation of initiator versus effector caspases, hypoxia/GSD primarily led to activation of caspase-8 in iPS-CMs (*P* = 0.03) but not in N-CMs (*P* = 0.3; Figure 9C). Compared to normoxic controls, caspase-9 activity did not change significantly in either iPS-CMs or N-CMs, while caspase-3/7 activity was similarly reduced in both cell types (iPS-CMs: *P* = 0.003; N-CMs: *P* = 0.003). With respect to advanced apoptotic or necrotic cell death with loss of plasma membrane integrity, 2 ± 0.16% of the iPS-CMs and 1.9 ± 0.09% of the N-CMs stained positive for 7-AAD in normoxic control medium (*P* = 0.47). In hypoxia/GSD, however, 10.3 ± 1.7% of the iPS-CMs displayed plasma membrane disruption with nucleotide staining by 7-AAD, while only 2.7 ± 0.06% of the N-CMs were 7-AAD-positive (*P* = 0.01; Figure 9B). By TUNEL staining, DNA fragmentation increased by 16.5 ± 0.8% in the iPS-CMs in response to hypoxia/GSD, but only by 2.5 ± 2.4% in the N-CMs (*P* = 0.001; Figure 9D). Since apoptotic cell death is preceded by breakdown of the mitochondrial membrane potential, we assessed the mitochondrial membrane potential using TMRM. As shown in Figure 10, TMRM accumulated in hyperpolarized mitochondria when iPS-CMs or N-CMs were subjected to normoxic control conditions. In hypoxia/GSD, TMRM fluorescence intensity was reduced by 35 ± 0.8% (*P* < 0.001) for iPS-CMs. This was not the case for N-CMs (reduction by 4.6 ± 0.8%; *P* = 0.47). Breakdown of mitochondrial membrane potential in many cells was also visualized using the fluorescent indicator JC-1. As shown in Figure 11, granular staining indicates mitochondrial JC-1 aggregates in hyperpolarized mitochondria when iPS-CMs or N-CMs were subjected to normoxic control conditions. In hypoxia/GSD, however, iPS-CMs displayed a diffuse staining pattern, indicating a significant reduction of the mitochondrial membrane potential in 35 ± 2% iPS-CMs. Again, this was not the case for N-CMs (0.37 ± 0.3%; *P* < 0.001; Figure 11B). ROS are known to mediate much of the cellular damage done during actual and simulated ischemia. ROS fluorescence intensity increased by 16.4 ± 2.5% (*P* = 0.005) in “ischemic” iPS-CMs in comparison to iPS-CMs cultured in normoxia, while ROS fluorescence intensity in “ischemic” N-CMs was increased by 7.1 ± 2% (*P* = 0.1; Figure 12B). In normoxia, significant differences in oxidative stress between iPS-CMs and N-CMs were not detected (*P* = 0.24).

### Cytoprotective effect of mesenchymal stromal cell-conditioned medium

**Figure 13:**
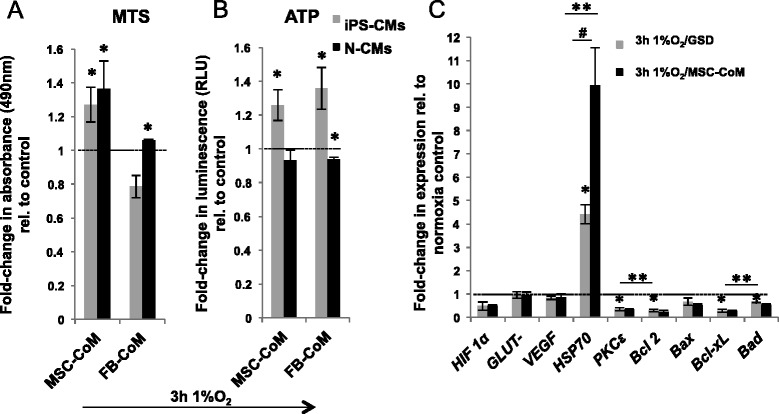
Effect of mesenchymal stromal cell-conditioned medium (MSC-CoM) and fibroblast-conditioned medium (FB-CoM) on metabolic activity and gene expression in hypoxia. **(A)** MSC-CoM and FB-CoM effects on the activity of cytosolic and mitochondrial oxidoreductases determined by MTS assay, and **(B)** MSC-CoM and FB-CoM effects on ATP content in induced pluripotent stem cell-derived cardiomyocytes (iPS-CMs) and neonatal cardiomyocytes (N-CMs). Data are normalized for respective average of hypoxia and glucose/serum deprivation (GSD) control experiments without MSC-CoM. *P ≤ 0.05, versus hypoxia/GSD controls. **(C)** Effect of hypoxia on expression of stress response genes in iPS-CMs incubated in standard or MSC-CoM in hypoxia/GSD. Data are shown as fold-change relative to the expression in normoxia/full medium (=1). *P ≤ 0.05, versus normoxia/full medium; ^#^ *P* ≤ 0.05, control versus MSC-CoM. **P ≤ 0.05, overall by analysis of variance.

To determine whether paracrine factors secreted by MSCs can counteract the increased susceptibility of iPS-CMs to hypoxia/GSD, cells were cultured in the presence of MSC-CoM or equivalent non-conditioned, glucose/serum-deprived control medium. To test whether medium conditioned by standard fibroblasts (FB-CoM) exerts similar effects, cells were also subjected to hypoxia/GSD in the presence of FB-CoM. As determined by MTS assay, MSC-CoM enhanced the oxidoreductase activity of iPS-CMs and N-CMs 1.27 ± 0.1-fold and 1.36 ± 0.17-fold, respectively (iPS-CM, *P* = 0.01; N-CMs, *P* = 0.05; Figure 13A), while FB-CoM had a less pronounced effect on oxidoreductase activity (iPS-CMs: *P* = 0.01; N-CMs: 1.05 ± 0.01-fold, *P* = 0.0001). In iPS-CMs, MSC-CoM also led to increased cytosolic ATP levels after 3 hours hypoxia/GSD (1.26 ± 0.1-fold, *P* = 0.009), and this effect was also observed in the presence of FB-CoM (1.35 ± 0.12-fold, *P* = 0.01), but not in N-CMs (MSC-CoM; *P* = 0.27, FB-CoM: *P* < 0.001; Figure 13B).

### Signaling response of induced pluripotent stem cell-derived cardiomyocytes to mesenchymal stromal cell-conditioned medium

**Figure 14:**
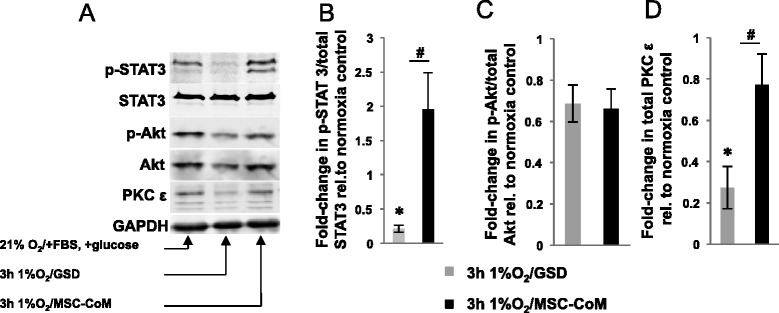
Western blot analysis of STAT3, p-STAT3, Akt, p-Akt and PKCε. **(A)** Representative immunoblots of STAT3, p-STAT3, Akt, p-Akt and PKCε expression in induced pluripotent stem cell-derived cardiomyocytes in normoxia/full medium (21% O_2_, + fetal bovine serum (FBS) + glucose), in hypoxia and glucose/serum deprivation (GSD) (1% O_2_; −FBS, −glucose), and in hypoxia/GSD with mesenchymal stromal cell-conditioned medium (MSC-CoM) (1% O2; +MSC-CoM). **(B-D)** Corresponding densitometry data of hypoxia/GSD with and without MSC-CoM, expressed as the fold-change compared to normoxia/full medium data. *P ≤ 0.05, versus normoxia/full medium; ^#^ *P* ≤ 0.05, versus MSC-CoM.

As assessed by real-time PCR, *Hif1*α, *Glut-1*, and *VEGF* transcripts were not significantly changed following hypoxia/GSD, and MSC-CoM did not induce the expression of these genes either (Figure 13C). The expression of mRNA encoding for *PKC*ε (*P* = 0.01), antiapoptotic genes *Bcl-2* (*P* = 0.05) and *Bcl-xL* (*P* = 0.003), and proapoptotic *Bad* (*P* = 0.02) was significantly reduced in cells exposed to hypoxia/GSD, while transcripts of the proapoptotic *Bax* gene were not affected. The expression ratios of *Bax/Bcl-2* and *Bad/Bcl-xL*, which are considered to be more reliable indicators of apoptosis, increased by 2.41 ± 0.67-fold (*Bax/Bcl-2*, *P* = 0.053) and 2.34 ± 0.23-fold (*Bad/Bcl-xL*, *P* = 0.59) in hypoxia/GSD. The presence of MSC-CoM had no influence on the expression of the above-mentioned genes. However, iPS-CMs exposed to hypoxia/GSD displayed significantly upregulated *Hsp70* mRNA levels (*P* = 0.04) and MSC-CoM further increased its expression (*P* = 0.006, versus normoxic control; *P* = 0.049, versus hypoxia/GSD). Proteins mainly regulated by phosphorylation were quantified by Western blotting with and without phospho-specific antibodies. Here, hypoxia/GSD significantly reduced the p-STAT3/STAT3 ratio in iPS-CMs (*P* = 0.004), while MSC-CoM efficiently preserved STAT3 phosphorylation (*P* = 0.03; Figure 14). Total PKCε was reduced in cells exposed to hypoxia/GSD (*P* = 0.01), but was restored to near-normal levels in the presence of MSC-CoM (*P* = 0.05). The p-Akt/Akt ratio did not change significantly during hypoxia/GSD and was not affected by co-incubation with MSC-CoM (*P* = 0.8).

## Discussion

So far, somatic stem cell therapy for heart repair has yielded disappointing clinical results [21], mainly because the capacity of non-pluripotent progenitors to re-create contractile cells upon transplantation in the failing heart has been overestimated. In this context, induced pluripotent stem cell technology offers the possibility to create large numbers of syngeneic CMs for potential clinical transplantation in the failing heart. Although there are concerns regarding the genetic stability of reprogrammed cells and their progeny [22,23], there are currently few, if any, conceptually sound alternatives for re-generation of contractile myocardium. iPS-CMs where previously shown to possess all the baseline phenotypic and functional characteristics of both CMs derived from embryonic stem cells and those isolated from neonatal myocardium [8-10,24,25], but little is known about their behavior in a hostile environment as it is encountered in a diseased heart.

We found that exposure to hypoxia combined with GSD,as a model of “simulated *in vitro* ischemia” led within 3 hours to caspase activation in almost 50% of the iPS-CMs, associated with an increased proportion of necrotic and TUNEL-positive cells, breakdown of the mitochondrial membrane potential in one third of the cells, increased accumulation of ROS, and a reduction of the average oxidoreductase activity by more than 60%. These findings mirror a substantial cell damage and are in line with studies showing that dying cells display characteristics of apoptosis, autophagy and necrosis at the same time [26]. Overall, N-CMs appeared to be less sensitive to these conditions because they exhibited less caspase activation, a minimal increase in the percentage of necrotic, TUNEL-positive cells and ROS expression, preserved mitochondrial membrane potential and a delayed and less pronounced loss of oxidoreductase activity. In line with these findings, caspase-8, which is an essential component of the extrinsic cell death pathways, was activated only in iPS-CMs but not in murine N-CMs after 3 hours hypoxia/GSD. Chao and colleagues showed that rat N-CMs activated caspase-8 after 4 hours exposure to hypoxia and serum deprivation [27], so that our finding of a 3-hour period of relative resistance to hypoxia/GSD in murine N-CMs appears realistic. On the other hand, caspase-9 activity did not increase and caspase-3 activity was reduced in both iPS-CMs and N-CMs. The initiator caspase-9 is a key component of the mitochondrial death pathway and would be expected to play an even greater role in cell death secondary to hypoxia/GSD [18,28,29]. This intrinsic apoptosis-initiating pathway is typically initiated by opening of the mitochondrial permeability pore when oxidative metabolism breaks down, with the latter being confirmed by our findings regarding the mitochondrial membrane potential. However, apoptosis in “ischemia-like” situations may also occur independently of the intrinsic pathway in a process involving “extrinsic” caspase-8 activation. For instance, Schamberger and colleagues demonstrated that in rat embryonic cells caspase-8 seemed to be sufficient to initiate the serum starvation-induced apoptosis while the caspase-9 activation was impaired by sequestration of caspase-9 to cytoskeletal structures [30]. Our finding of downregulated effector caspase-3/7 activity in both iPS-CMs and N-CMs underscores the relatively early stage of apoptosis induction in the majority of the cells or the non-apoptotic functions that these caspases may exert under these conditions, as has been described for several other cellular systems [31-33].

The key question is whether the increased susceptibility to hypoxia/GSD compared to N-CMs is a phenomenon caused by reprogramming, or reflects differences in CM maturity. As Keung and colleagues have recently summarized [9], controlling iPS-CM maturation is exceedingly important in order to provide a uniform cell product with stable characteristics for therapeutic or advanced research applications. We used cells collected at differentiation day 21, which are expected to have matured beyond the fetal stage. However, several of the assays we performed regarding calcium handling, cytoplasmic membrane potential and mitochondrial respiratory function indicate that at least a proportion of our iPS-CM population, although produced and purified using a highly standardized protocol, are more immature. Calcium transients of the iPS-CMs resemble calcium transients observed in primary neonatal mouse CMs [34] or mouse embryonic stem cell-derived cardiomyocytes (ESC-CMs) [35]. However, compared with primary adult mouse CMs [36,37], calcium transients of iPS-CMs had reduced systolic maximal calcium as well as rates of both fluorescence rise during systole and decay during diastole. Electrophysiological data indicate that iPS-CMs display properties as described previously for fetal CMs and ESC-CMs [10,38,39] and the Seahorse data suggest that iPS-CMs exhibit an active metabolic profile that is comparable to that reported for human iPS-CMs [40] and murine N-CMs [41-43]. Nevertheless, the comparison with N-CMs appears justified to us, since many functional and morphologic similarities have been previously reported and were also confirmed in our study. In the end, individual CMs developing in an artificial *in vitro* setting may display differing degrees of maturity, so that uniformity would be reached only when all cells have completely matured. Of note, Boheler and colleagues recently showed that the viability of ESC-CMs obtained at differentiation day 10 decreased as late as after 48 hours exposure to 0.5% O_2_ and serum and glucose deprivation [7]. At that point, more than 70% of the immature ESC-CMs were still viable, while late-stage ESC-CMs obtained on differentiation day 18 were much more sensitive to “simulated ischemia”. It has been well documented that CMs isolated from fetal hearts are less sensitive to hypoxia/ischemia than those from neonatal hearts [44]. Comparison with mature/adult CMs is problematic because naïve adult CMs are difficult to culture, but intact neonatal hearts have been shown to be more sensitive to ischemia than mature hearts [45]. Nevertheless, our data allow us to conclude that murine iPS-CMs created with this well-established protocol, which has been used in numerous experimental settings [14,46,47], have a distinct deficit regarding their tolerance to “simulated ischemia” that needs to be taken into account when interpreting outcome data and requires countering measures.

One such measure may be the preceding or concomitant incubation with MSCs or, as in our experiments, with MSC-CoM. We have shown previously that MSC-CoM increases the hypoxia/GSD tolerance of the CM-like HL-1 cells [48], and other groups demonstrated the efficacy of this approach in intact ischemic hearts [49,50]. MSC-CoM increased the cellular redox activity in iPS-CMs subjected to hypoxia/GSD approximately 1.3-fold, a modest but possibly relevant improvement which might be further enhanced by concentrating the medium or adding live MSCs [14]. FB-CoM did only partially mimic the cytoprotective effects of MSC-CoM, which emphasizes the ‘beneficial’ specificity of MSC-released paracrine factors. On the molecular level, our model did not induce mRNA expression of hypoxia-inducible genes such as *Hif1*α, *Vegf* and *Glut-1*, but Hsp70 transcription was upregulated and further augmented in the presence of MSC-CoM. This is in line with other studies showing that heat shock proteins support the survival of cells exposed to environmental stress [51-53], as well as with reports showing that HSP70 promotes CM survival by inhibiting pro-apoptotic pathways that include caspase- and Fas-mediated death cascades [54]. Furthermore, *Bcl-2*, *Bcl-xL* and *Bad* transcripts were reduced in hypoxia while the *Bax/Bcl-2* and *Bad/Bcl-xL* ratio in hypoxia increased, which agrees with the increased caspase activity that we detected. Regarding the canonical kinase signaling pathways, MSC-CoM in our study prevented reduction in PKCε protein levels and restored STAT3 phosphorylation to almost basal levels, confirming the activation of cell survival pathways by paracrine factors that were also shown to mediate the MSC-CoM response in other myocyte cell types [48].

### Limitations

The simulated ischemia model we used in these *in vitro* experiments is clearly only a rudimentary representation of the situation present in an ischemic heart. Moreover, we concentrated on the damage induced by “simulated ischemia” alone but did not perform experiments that mimic reperfusion/reoxygenation injury. Also, our iPS-CM population was genetically modified and purified by antibiotic selection, while the N-CMs we used were naïve cells. While N-CM purity was originally confirmed to be high, it cannot be ruled out that during longer cultivation periods the proportion of proliferative non-CM cells increased.

## Conclusions

In summary, we demonstrated that 3 hours hypoxia and GSD provoke cell stress and damage in iPS-CMs that exceeds that of naïve N-CMs, although the iPS-CMs we used showed several aspects of less mature CMs, which are considered to be more resistant to hypoxia and/or ischemia. While the reasons for this increased susceptibility are not entirely clear, a translationally practical approach to influence it may be the concomitant treatment with MSC-CoM via *HSP70* expression, preservation of PKCε-dependent signaling cascades and further activation of STAT3-dependent signaling cascades. Ultimately, this strategy may help improve the efficacy of regenerative therapies involving CMs derived from iPS cells.

## Note

 This article is part of a thematic series on *Cardiovascular regeneration* edited by Ronald Li. Other articles in the series can be found online at http://stemcellres.com/series/cardiovascular.

## Abbreviations

7-AAD: 7-aminoactinomycin D
AP: action potential
APD90: action potential duration at 90% of repolarization
BSA: bovine serum albumin
CM: cardiomyocyte
DMEM: Dulbecco’s modified Eagle's Medium
EB: embryoid body
EDTA: ethylenediaminetetraacetic acid
ESC-CM: embryonic stem cell-derived cardiomyocyte
FB-CoM: fibroblast-conditioned medium
FBS: fetal bovine serum
FCCP: carbonyl cyanide-4-(trifluoromethoxy)phenylhydrazone
GFP: green fluorescent protein
GSD: glucose/serum deprivation
HBSS: Hank’s balanced salts modified buffer
HCS: high content screening
IMDM: Iscove’s modified Dulbecco’s medium
iPS: induced pluripotent stem cell
iPS-CM: induced pluripotent stem cell-derived cardiomyocyte
MSC: mesenchymal stromal cell
MSC-CoM: mesenchymal stromal cell-conditioned medium
N-CM: neonatal cardiomyocyte
OCR: oxygen consumption rate
PBS: phosphate-buffered saline
PFA: paraformaldehyde
ROS: reactive oxygen species
TMRM: tetramethylrhodamine methyl ester
